# Staged Therapeutic Approach to Concomitant Severe Aortic Stenosis and Atrial Septal Defect: Addressing Dual Mechanisms of Heart Failure—Case Report and Literature Review

**DOI:** 10.3390/life16081360

**Published:** 2026-08-19

**Authors:** Crina-Ioana Radulescu, Catalina-Andreea Parasca, Roxana Enache, Teodora Maria Barboi, Nicu Catana, Dan Deleanu, Pavel Platon, Serban Bubenek-Turconi, Vlad Anton Iliescu

**Affiliations:** 1 Department of Cardiovascular Surgery, “Prof. Dr. C.C. Iliescu” Emergency Institute for Cardiovascular Diseases, 022328 Bucharest, Romania; 2 Faculty of Medicine, Carol Davila University of Medicine and Pharmacy, 050474 Bucharest, Romania; 3 Department of Interventional Cardiology, “Prof. Dr. C.C. Iliescu” Emergency Institute for Cardiovascular Diseases, 022328 Bucharest, Romania; 4 Department of Anesthesiology and Intensive Care, “Prof. Dr. C.C. Iliescu” Emergency Institute for Cardiovascular Diseases, 022328 Bucharest, Romania

**Keywords:** aortic stenosis, pulmonary hypertension, atrial septal defect

## Abstract

Background: Severe aortic stenosis (AS) remains the most prevalent primary valvular disease in Europe and North America, causing heart failure (HF), with pulmonary hypertension (PH) occurring in up to 75% of symptomatic patients. The coexistence of AS and atrial septal defect (ASD) is rare and may generate dual mechanisms of PH, complicating both diagnosis and management. Case summary and review: We report a 78-year-old patient with symptomatic severe AS and right heart failure, in whom an unrecognized secundum ASD with significant left-to-right shunt was identified as a major contributor to persistent HF and right ventricular (RV) dysfunction. Due to prohibitive surgical risk, a staged interventional strategy was decided: transcatheter aortic valve implantation (TAVI), followed by cardiac catheterization and ultimately by percutaneous ASD closure. Marked clinical benefit with the reduction of the RV dimensions and improved systolic function were observed at one year follow-up. A focused review of the literature was conducted to contextualize the pathophysiological mechanisms, diagnostic challenges, and therapeutic strategies in patients with coexisting AS and ASD. Conclusions: This case emphasizes the importance of comprehensive hemodynamic assessment in AS complicated by PH and HF, particularly in the presence of congenital anomalies such as ASD.

## 1. Introduction

Severe aortic stenosis (AS) is the most common valvular heart disease requiring intervention in developed countries, with its prevalence steadily increasing due to population aging [1]. Pulmonary hypertension (PH) complicates more than half of symptomatic AS cases and is a well-established marker of poor prognosis [2,3], largely mediated by elevated left-sided filling pressures, progressive left and right ventricular (RV) dysfunction and subsequent HF.

The coexistence of severe AS and an atrial septal defect (ASD) is exceptionally uncommon and results in complex, multifactorial hemodynamic interactions. In such patients, PH often develops through combined mechanisms: a postcapillary component secondary to elevated left ventricular (LV) filling pressures due to pressure overload from AS, and an additional contribution from volume overload related to a significant left-to-right interatrial shunt. This dual burden of pressure and volume overload exerts a synergistic effect on pulmonary circulation and RV remodeling, influencing both the reversibility of PH and the optimal sequencing of therapeutic interventions [4,5].

Moreover, in such cases, the onset of AS-related symptoms may be delayed or attenuated because the interatrial shunt can temporarily decompress the left atrium (LA), serving as a partial “relief mechanism” that mitigates the hemodynamic impact of increased LV filling pressures [6]. Consequently, the natural history and clinical presentation of AS in these patients may be atypical or masked, underscoring the importance of early and comprehensive echocardiographic evaluation to identify such coexisting lesions before advanced decompensation occurs [1,7].

Therefore, we present a challenging case of severe AS with an unrecognized ostium secundum ASD, manifesting with severe PH, RV dysfunction and congestive HF. This case illustrates the need for a multidisciplinary, staged approach combining transcatheter aortic valve implantation (TAVI), heart failure–directed medical therapy, and percutaneous ASD closure.

## 2. Case Presentation

### 2.1. Patient Presentation

This is the case of a 78-year-old man with a recent diagnosis of severe AS who presented with progressive signs of congestive heart failure. The patient’s baseline clinical profile was characterized by moderate frailty (Clinical Frailty Score 6) and multiple comorbidities, namely permanent atrial fibrillation, type 2 diabetes mellitus and dyslipidemia. His at home treatment consisted in low dose beta-blocker (bisoprolol 2.5 mg od), ACE inhibitor (ramipril 2.5 mg od), direct oral anticoagulant (apixaban 5 mg, bid) and statin (atorvastatin 20 mg od). On physical examination, he was tachypneic (respiratory rate 30/min) and hypoxemic (SpO_2_ 88% on room air). Physical findings were consistent with both systemic and pulmonary congestion, including elevated jugular venous pressure, hepatomegaly, bilateral pitting edema, basal crackles, and bilateral pleural effusions.

Laboratory evaluation revealed a markedly elevated NT-proBNP (12,896 pg/mL), positive high-sensitivity troponin without significant dynamic changes (between 30 and 43 ng/L considering an upper reference limit of 29 ng/L), and mild normocytic anemia. Furthermore, the patient had preserved renal function with a creatinine of 0.89 mg/dL, but presented mild hepatocytolysis and cholestatic syndrome (ALT 75 U/L, AST 51 U/L, total bilirubin 2.6 mg/dL with a direct bilirubin of 1.35 mg/dL). The 12-lead ECG demonstrated atrial fibrillation with a ventricular rate of 110 bpm, QRS duration of 124 ms and poor R-wave progression in the precordial leads.

Point-of-care transthoracic echocardiography (TTE) performed in the emergency department revealed a non-dilated left ventricle with mildly reduced ejection fraction, severe aortic stenosis (peak velocity 4.5 m/s), and moderate secondary mitral regurgitation. Additionally, there was severe tricuspid regurgitation, marked right cavities dilatation and RV dysfunction, with echocardiographic signs of PH.

After prompt stabilization in the emergency department with low-dose intravenous furosemide boluses (40 mg) and supplemental oxygen therapy, the patient was admitted to the Cardiology Department for further hemodynamic assessment and management.

### 2.2. Diagnostic Work-Up

Upon admission to the Cardiology Department, intravenous loop diuretic therapy was initiated at a dose 2.5 times higher than the patient’s home regimen. However, within the first 24 h, the patient failed to achieve an adequate natriuretic response (spot urine sodium: 40 mEq/L). Consequently, the loop diuretic dose was titrated up to 200 mg/day, and a low-dose thiazide (hydrochlorothiazide 25 mg) was added, resulting in a negative sodium balance and a significant improvement in diuresis and clinical congestion [8].

**Figure 1:**
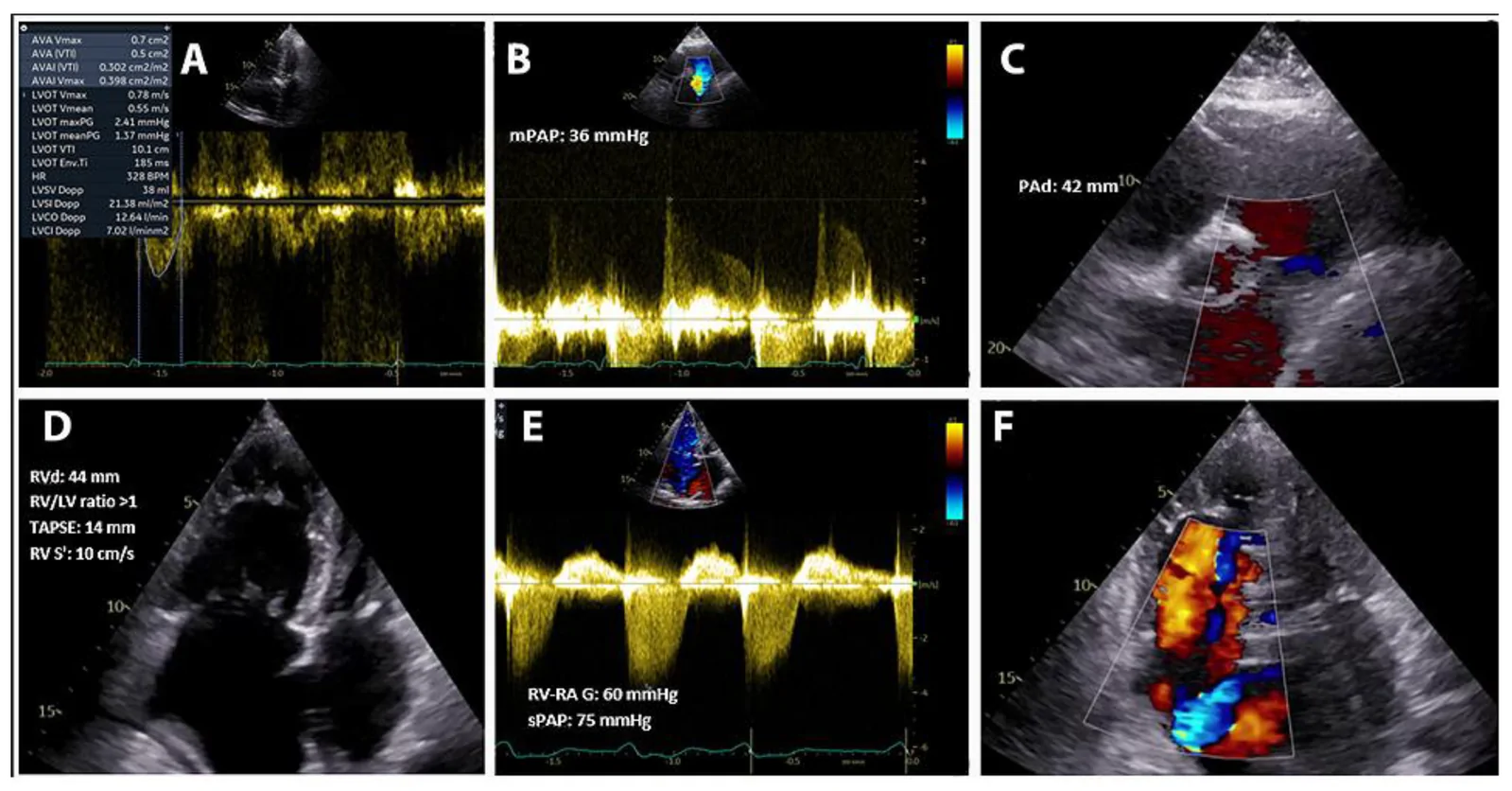
**Post-decongestion echocardiographic reassessment.** (**A**) Degenerative aortic valve disease with severe aortic stenosis (Vmax 4.7 m/s, mean gradient 45 mmHg, AVA 0.53 cm^2^, AVA index 0.29 cm^2^/m^2^) and mild regurgitation; (**B**) High probability of pulmonary hypertension (non-invasive mean pulmonary artery pressure of 36 mmHg determined using CW doppler early diastolic velocity gradient); (**C**) Moderate pulmonary regurgitation with dilatation of the pulmonary artery (PA diameter 42 mm); (**D**) Dilated right ventricle (RV diameter 44 mm, RV/LV ratio >1) with significant RV systolic dysfunction (TAPSE 14 mm, RV s′ 10 cm/s); (**E**) Severe pulmonary hypertension, demonstrated by consistently increased RV-RA gradient; (**F**) Severe tricuspid regurgitation on color doppler interrogation.

After five days of effective decongestive therapy, a comprehensive transthoracic echocardiography was performed (Figure 1), demonstrating severe AS (Vmax: 4.7 m/s, mean gradient: 45 mmHg, calculated AVA of 0.53 cm^2^, AVA index: 0.29 cm^2^/m^2^, and a dimensionless index of 0.17) and mild LV systolic dysfunction (LVEF: 45%, VTI LVOT: 10 cm with an indexed stroke volume of 22 mL/m^2^). The interventricular septum showed systolic–diastolic flattening consistent with RV pressure–volume overload. There was severe RV dilatation with significant systolic dysfunction (TAPSE: 14 mm, RV S′: 10 cm/s), and severe tricuspid regurgitation, with an estimated systolic pulmonary artery pressure (sPAP) of 75 mmHg after decongestion. Even if severe tricuspid regurgitation and RV dysfunction may underestimate the severity of PH, consistently increased RV-RA gradients and the dilated inferior vena cava confirm the elevated systolic pulmonary pressures. Moreover, the mean pulmonary artery pressure measured on the pulmonary regurgitation CW doppler assessment (36 mmHg) is independent of RV function or the degree of tricuspid regurgitation. Given the disproportionate right heart chambers dilatation and PH severity (pulmonary artery diameter 42 mm, RV/LV ratio >1), a noninvasive shunt quantification was performed, using the standard Doppler method based on stroke volume measurements across the pulmonary and systemic outflow tracts, yielding a Qp/Qs ratio of 3.9, strongly suggesting the presence of a significant intracardiac left-to-right shunt.

**Figure 2:**
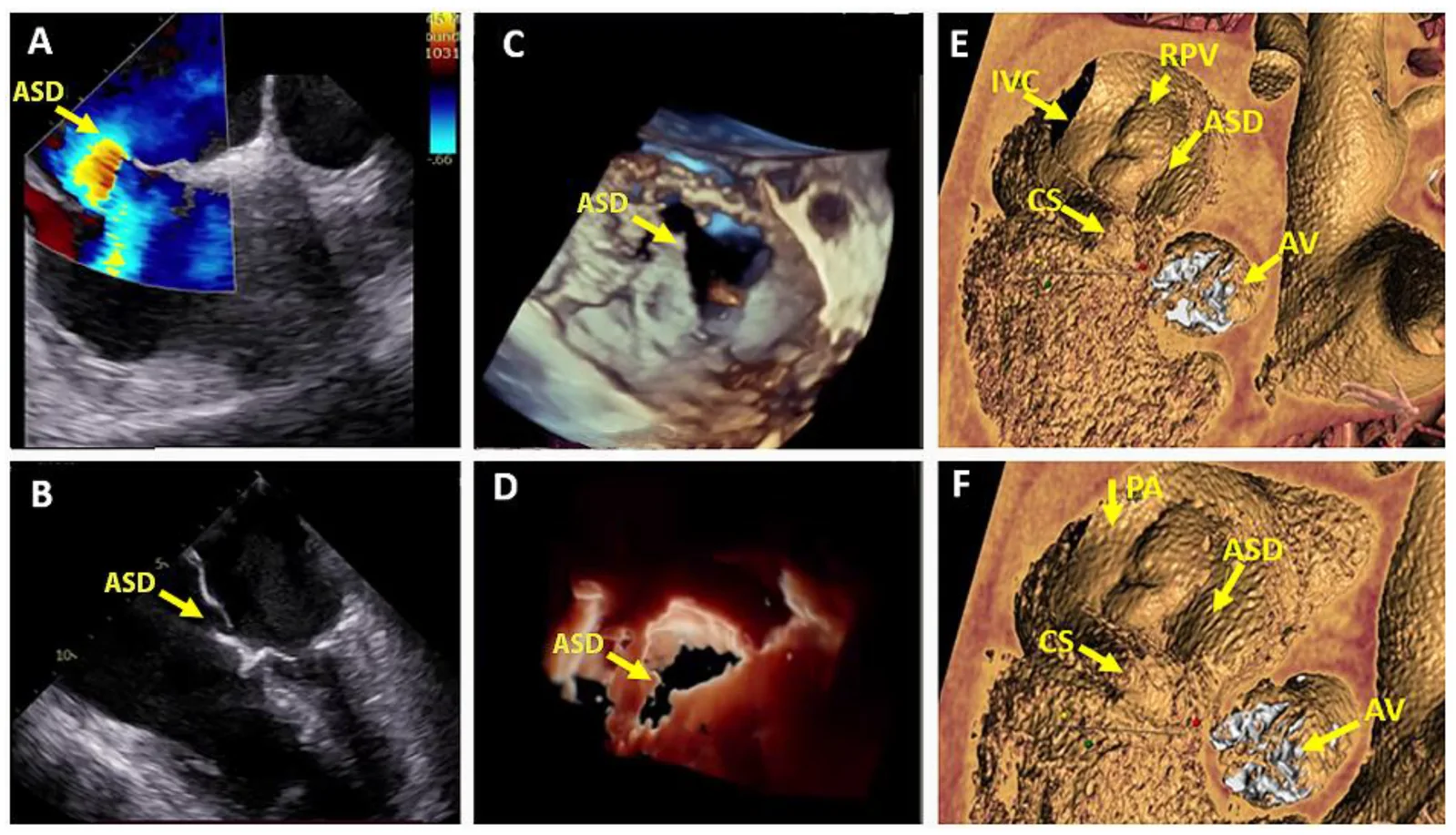
**Multimodal imaging evaluation of a left-to-right interatrial shunt.** (**A**) TOE—modified bicaval view (100°) demonstrating a large ASD (21 × 21 mm) with left-to-right flow on color Doppler imaging; (**B**) Zero-degree TOE view showing the hemodynamic impact of the ASD, with marked right heart chamber dilatation and abnormal septal mobility; (**C**,**D**) TOE reconstruction illustrating the specific anatomy of the defect, characterized by a small posterior rim and multiple fenestrations consistent with a cribriform septum; (**E**,**F**) Cardiac CT demonstrating normal pulmonary venous drainage, a large ostium secundum ASD, and a tricuspid aortic valve with degenerative, calcified cusps.

Initial TTE did not clearly delineate the defect; therefore, transesophageal echocardiography (TOE) was performed for a more detailed anatomic assessment (Figure 2A–D). This revealed a large atrial septal defect (21 × 21 mm) with a complex cribriform morphology, multiple fenestrations, and a small posterosuperior rim, which was visualized using three-dimensional reconstruction. To further assess cardiac anatomy, exclude other associated congenital defects, and confirm normal pulmonary venous return, cardiac computed tomography (CT) was performed. The CT confirmed the presence of a large cribriform ostium secundum ASD with normal pulmonary venous drainage and no additional abnormalities (Figure 2E,F). It is worth noting that after effective decongestion, guideline directed medical therapy (GDMT) was optimized by adding mineralocorticoid receptor antagonist and SGLT2 inhibitor whilst maintaining the doses of beta-blocker and ACE inhibitor.

### 2.3. Management Strategy

After this comprehensive multimodality imaging evaluation, the case was discussed within the institutional Heart Team. Given the patient’s severe symptomatic aortic stenosis, advanced age, frailty and high surgical risk (EuroSCORE II: 8.45%), the consensus was to proceed with TAVI [1,5], followed by reassessment of clinical and hemodynamic status to determine the optimal timing for ASD closure.

**Figure 3:**
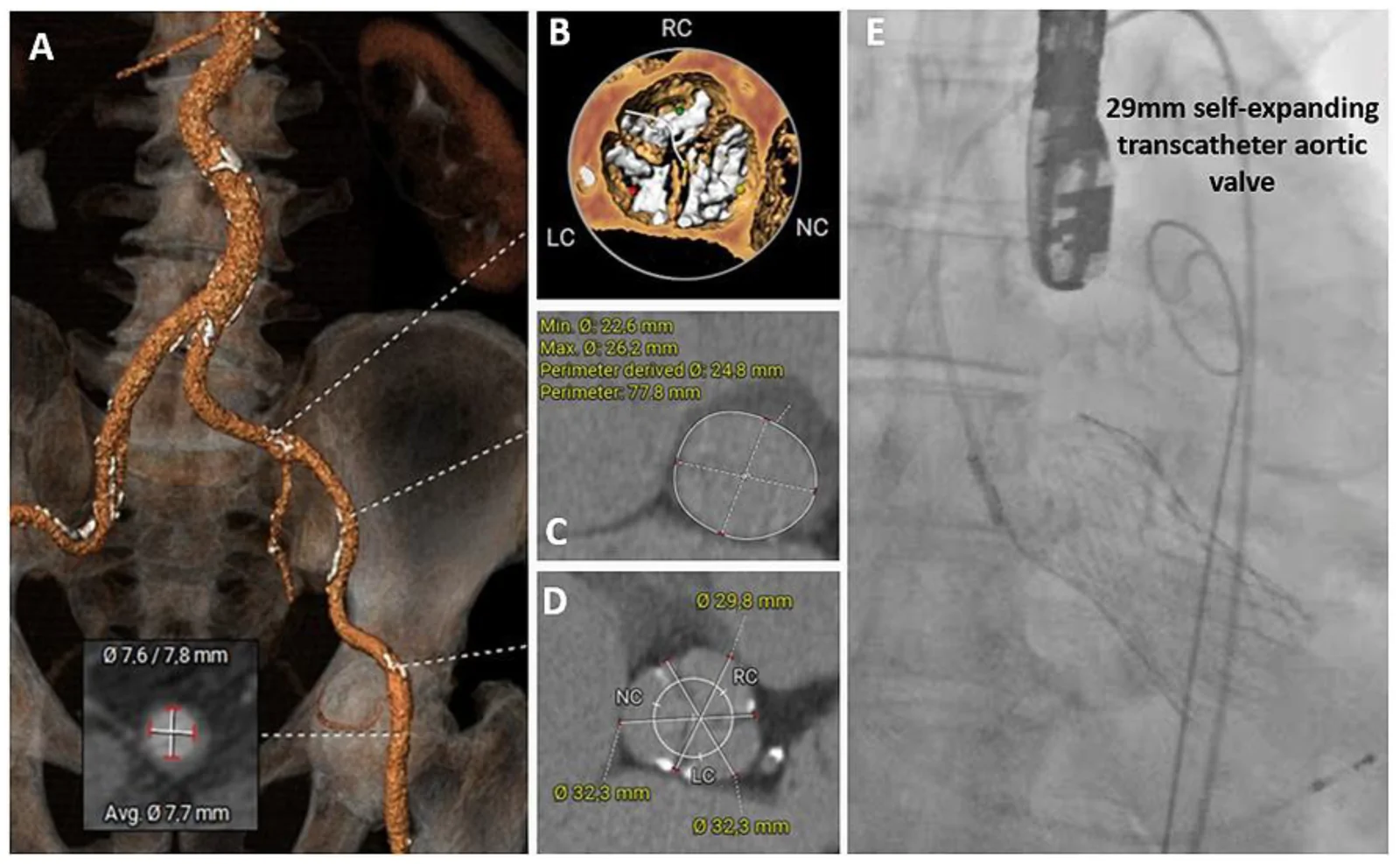
**Pre-procedural multidetector CT and procedural imaging assessment for TAVI planning and implantation.** (**A**) Favorable iliofemoral access; (**B**) Tricuspid, heavily calcified aortic valve; (**C**) Aortic annulus with a mean diameter of 24.4 mm, perimeter of 77.8 mm, and area of 476.2 mm^2^; (**D**) Favorable aortic root anatomy; (**E**) Successful implantation of a 29-mm self-expanding transcatheter aortic valve under combined fluoroscopic and TOE guidance.

Pre-procedural multidetector CT confirmed favorable iliofemoral access and anatomical suitability for transcatheter valve implantation (Figure 3A–D). The aortic annulus had a mean diameter of 24.4 mm, a perimeter of 77.8 mm, and an area consistent with optimal sizing for a 29-mm self-expanding valve. The LV outflow tract area measured 480 mm^2^. The aortic root anatomy was favorable, with sinus of Valsalva dimensions of 22.8 mm, 26.8 mm, and 23.0 mm for the left, right, and non-coronary cusps, respectively. The coronary ostial heights were 17.4 mm for the left and 22.2 mm for the right coronary artery, indicating sufficient clearance from the annular plane and confirming procedural feasibility.

Under combined fluoroscopic and TOE guidance, a 29-mm self-expanding Evolut^TM^ FX transcatheter valve (Medtronic, Minneapolis, MN, USA) was implanted successfully (Figure 3E). The procedure was uneventful, with excellent valve positioning, no paravalvular leak, and immediate hemodynamic improvement.

Follow-up transthoracic echocardiography performed two days later demonstrated a normally functioning bioprosthesis (peak velocity: 1.6 m/s, mean transvalvular gradient: 6 mmHg, effective orifice area index: 0.93 cm^2^/m^2^). The LV showed mild systolic dysfunction (LVEF: 48%), likely reflecting the persistent burden of right-sided pressure overload. Notably, there was a rapid improvement in RV performance following afterload reduction, with TAPSE increasing to 16 mm and RV S′ velocity to 10 cm/s.

GDMT for HF was continued, including SGLT2 inhibition, beta-blocker, ACE inhibitor, mineralocorticoid receptor antagonist, and loop diuretics [8].

### 2.4. Follow-Up

**Figure 4:**
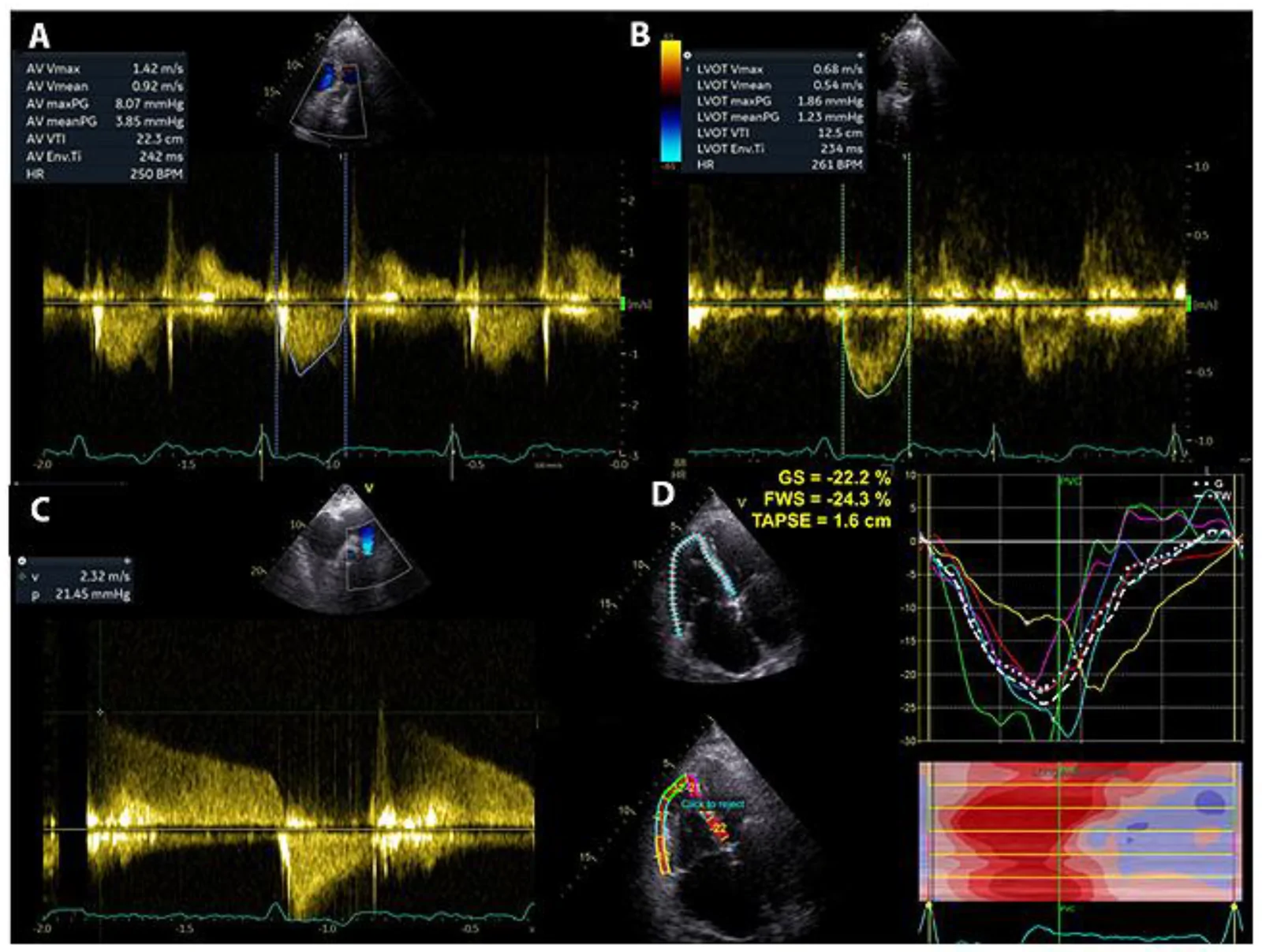
**TTE evaluation one month after TAVI.** (**A**,**B**) Normally functioning aortic bioprosthesis (peak velocity: 1.43 m/s, mean transvalvular gradient: 3.8 mmHg, effective orifice area index: 0.82 cm^2^/m^2^) without paravalvular leaks; (**C**) Persistent, though improved, PH (estimated mPAP: 21 mmHg); (**D**) Significant improvement in RV systolic performance (TAPSE: 16 mm, RV FWS: −24.3%).

At the 1-month follow-up, the patient showed marked clinical improvement, being able to walk 300 m on the six-minute walk test, with a significant reduction in NT-proBNP levels (890 pg/mL). TTE demonstrated a normally functioning aortic bioprosthesis and improved biventricular systolic performance (LVEF: 55%, RV fractional area change: 43%, RV FWS: −24%) (Figure 4). Despite this favorable remodeling, non-invasive Doppler assessment still indicated mild residual pulmonary hypertension (estimated mPAP: 21 mmHg).

**Figure 5:**
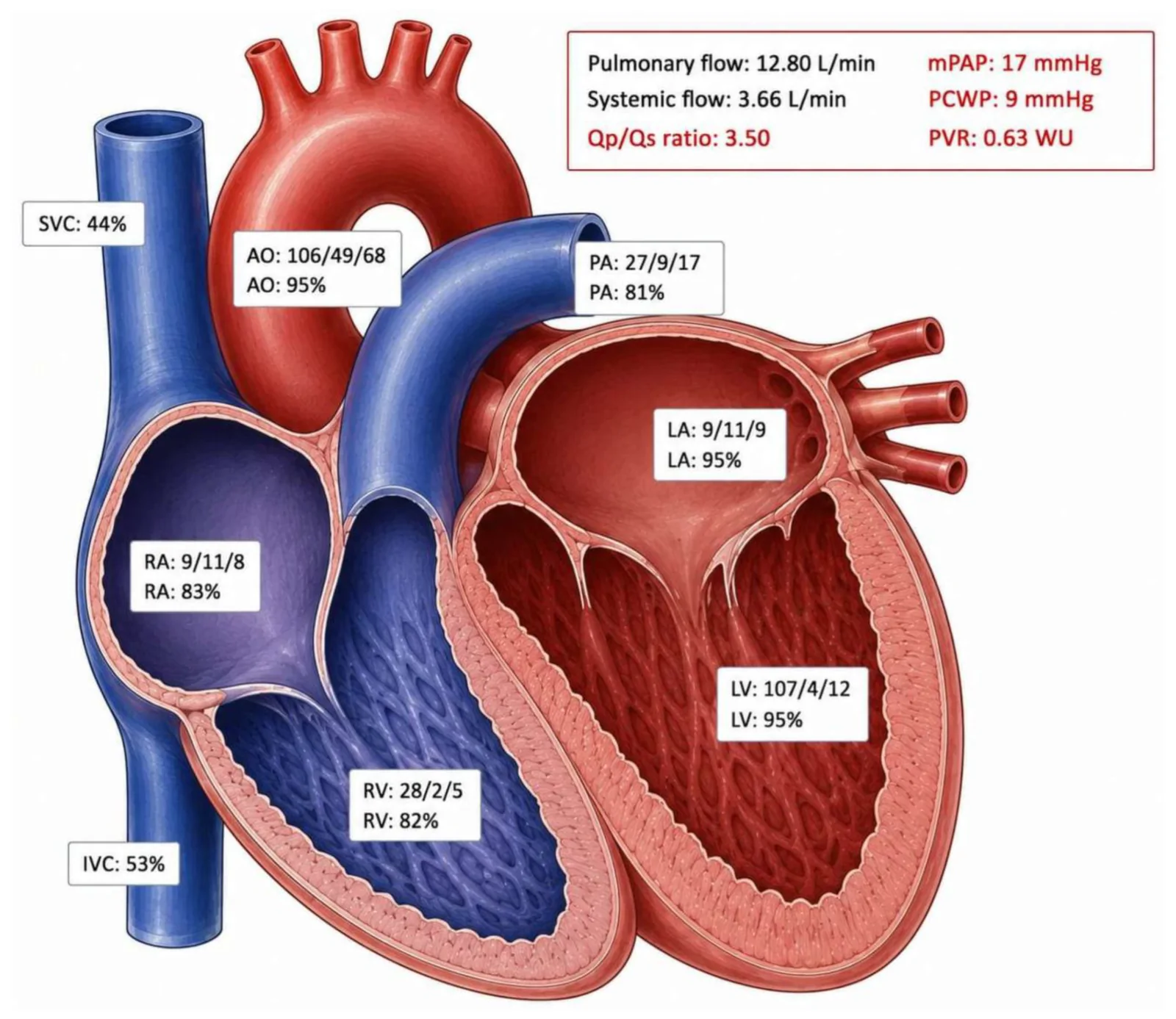
**Right heart catheterization**—performed after effective decongestion, optimization of GDMT and TAVI, confirming a significant left-to-right shunt (Qp/Qs: 3.5) and normalization of pulmonary pressures (mPAP: 17 mmHg, PCWP: 9 mmHg, PVR: 0.63 WU).

Given the patient’s euvolemic status and the absence of residual pressure overload following TAVI, it was considered an appropriate time to reassess the indication for ASD closure. Right heart catheterization (RHC) confirmed a significant left-to-right shunt (Qp/Qs 3.5), along with normalized pulmonary hemodynamics (mPAP: 17 mmHg, pulmonary capillary wedge pressure 9 mmHg, PVR 0.63 Wood units) (Figure 5). In this context, with persistent symptoms, evidence of RV volume overload, and in the absence of irreversible pulmonary arterial hypertension—the patient met a Class I, Level B indication for percutaneous ASD closure according to current guidelines [9].

**Figure 6:**
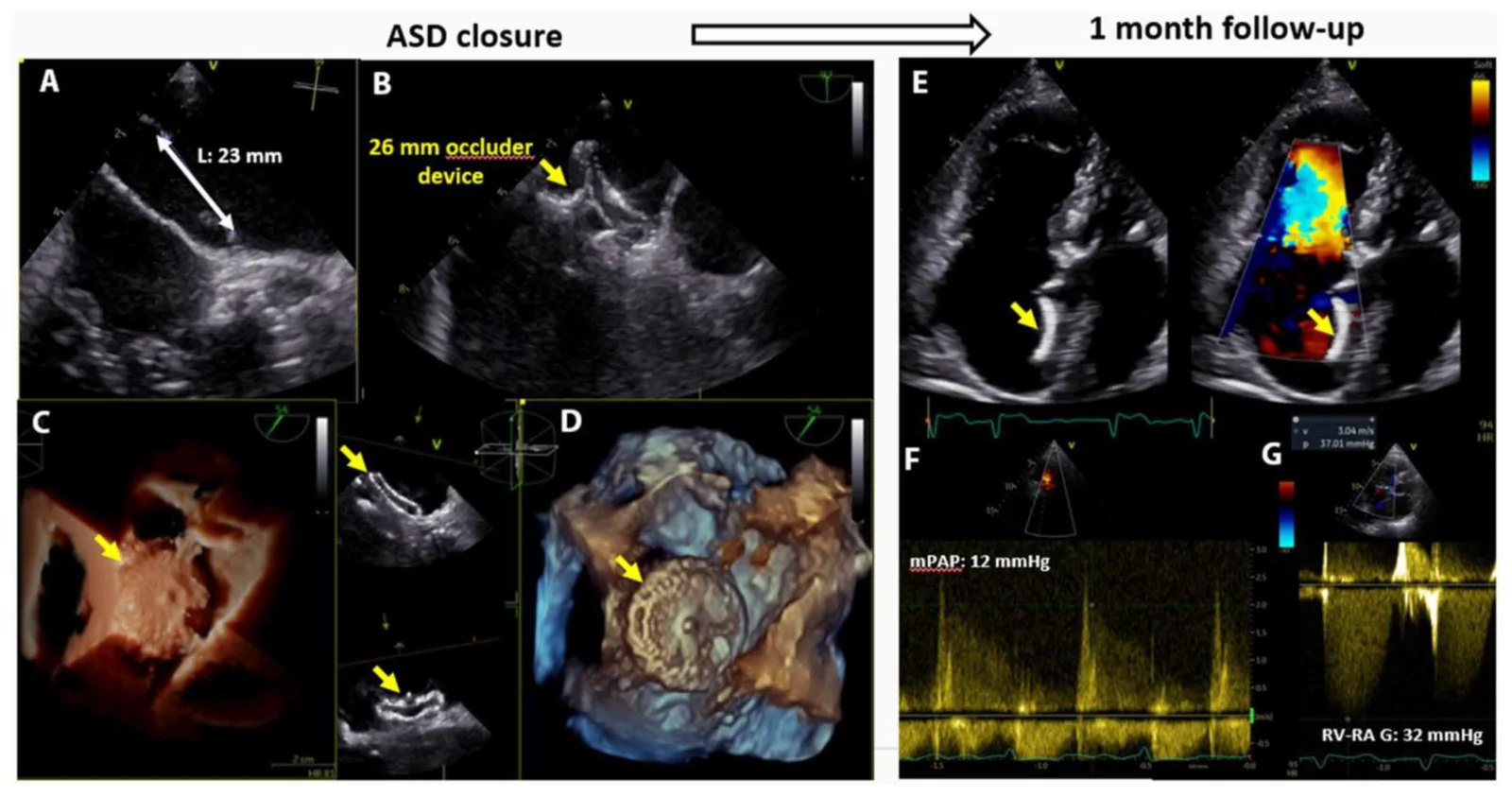
**Percutaneous atrial septal defect (ASD) closure and follow-up evaluation.** (**A**) Pre-procedural planning: modified bicaval TOE view showing a maximal ASD diameter of 23 mm; (**B**) Intraprocedural positioning of the occluder device; (**C**,**D**) Three-dimensional TOE echocardiographic reconstruction after successful deployment of a 26-mm occluder device; (**E**) Follow-up at 1 month demonstrating a well-seated device with a small residual shunt; (**F**,**G**) Significant improvement in PH (estimated mPAP: 12 mmHg).

TOE before the procedure confirmed a large secundum ASD with a maximum diameter of 23 mm and adequate surrounding tissue margins to securely anchor the closure device and prevent complications such as device embolization or erosion. All the key atrial rims (aortic, atrioventricular, postero-superior and postero-inferior) measured at least 5 mm. Anatomy was considered suitable for the implantation of a 26-mm Cocoon ASD occluder (Vascular Innovations Co., Ltd., Nonthaburi, Thailand) which was chosen by the operator, the device size being calculated by adding 2–4 mm to the maximum measured diameter (14–20% percentage of oversizing). The intervention was performed percutaneously under fluoroscopic and echocardiographic guidance, with successful device deployment, complete hemodynamic stability, and no procedural complications (Figure 6). The patient was discharged maintaining GDMT for HF. Considering the presence of permanent atrial fibrillation and frailty, only direct oral anticoagulation was decided as antithrombotic therapy.

At the 1-month follow-up, the patient exhibited further symptomatic improvement, remaining in NYHA functional class II and achieving 400 m on the six-minute walk test. Echocardiographic assessment revealed a well-seated occluder device with a small residual shunt, further improvement in RV volumes and systolic function, and a significant reduction in pulmonary pressures (estimated mPAP: 12 mmHg). On longer follow-up, at 6 months and one year, the patient exhibited improved functional capacity with no clinical signs of congestive HF.

## 3. Discussion

### 3.1. Severe Aortic Stenosis and Atrial Septal Defect—Epidemiology

ASD is among the most common congenital heart defects diagnosed in adulthood, accounting for approximately 10–15% of all congenital heart disease [10,11]. The prevalence of ASD in the general adult population is estimated at 0.88 per 1000, with secundum-type defects representing approximately 75% of cases [11]. However, ASD associated with severe AS represents a much rarer clinical entity, with an estimated prevalence of approximately 3–4% among adult patients with congenital or degenerative aortic valve disease, most often in the setting of bicuspid aortic valve or congenital aortic stenosis [12,13].

The present case is particularly remarkable for depicting an unrecognized, uncorrected ASD persisting into advanced age in a patient who later developed severe degenerative tricuspid aortic valve stenosis. Such coexistence of congenital and acquired lesions results in complex hemodynamic interactions that challenge both diagnosis and management. The natural history of uncorrected ASD includes progressive RV dilation, atrial arrhythmias, paradoxical embolism, and the eventual development of PH in approximately 6–35% of cases, with higher rates observed in patients with larger defects and those diagnosed after the fourth decade of life [11,14].

### 3.2. Pulmonary Hypertension—Pathophysiology of Dual-Mechanism

#### 3.2.1. Pathophysiology of PH

PH, reported in up to 75% of symptomatic AS patients [2,3], typically results from chronically elevated LV and LA pressures, generating a postcapillary hemodynamic profile. In the coexistence of severe AS and an ASD, two major determinants of postcapillary PH—pressure overload from AS and volume overload from the interatrial shunt—act synergistically. Their combined and sustained effect on pulmonary circulation may, over time, lead to vascular remodeling and the emergence of a precapillary component, thereby amplifying RV afterload and worsening overall prognosis [15,16].

The current hemodynamic classification of PH recognizes combined post- and pre-capillary pulmonary hypertension (CpcPH) as a distinct entity characterized by elevated mean pulmonary artery pressure (mPAP > 20 mmHg), elevated pulmonary capillary wedge pressure (PCWP > 15 mmHg), and elevated pulmonary vascular resistance (PVR ≥ 2 Wood units) [17,18]. This phenotype is associated with worse outcomes compared with isolated postcapillary PH, as it reflects more advanced pulmonary vascular remodeling and RV dysfunction [16,19]. In the present case, the initial hemodynamic profile was consistent with more significant postcapillary PH. The residual shunt-driven volume overload justified the subsequent ASD closure.

Notably, the interatrial shunt in this setting may serve as a paradoxical protective role by decompressing the LA, thereby attenuating the transmission of elevated LV filling pressures to the pulmonary circulation [20]. This phenomenon may partially explain the delayed clinical presentation in patients with coexisting AS and ASD, as the shunt mitigates pulmonary venous congestion at the expense of progressive RV volume overload. However, this compensatory mechanism is ultimately maladaptive, as sustained RV volume overload leads to progressive RV dilation, tricuspid annular dilatation, functional tricuspid regurgitation, and eventual RV failure [21,22].

#### 3.2.2. Pulmonary Vasculature and Tricuspid Damage

An interesting view is the staging of AS based on the extent of extravalvular cardiac damage on risk stratification first described by Genereux et al. [23], studying the patients in the PARTNER 2 trial [24]. The rationale was based on the pathophysiological alterations that the LV is first affected, followed by alterations in the LA pressure, pulmonary vasculature and finally RV dysfunction. Patients have been categorized as having no extra-aortic damage (stage 0), LV damage consisting in LV dysfunction or severe hypertrophy (stage 1), LA or mitral valve damage (stage 2), pulmonary vasculature defined by sPAP over 60 mmHg or moderate-to-severe tricuspid regurgitation (stage 3) and RV damage and moderate or severe dysfunction defined as TAPSE < 16 mm (stage 4) [25]. Interestingly enough, cardiac damage identified in patients was not necessarily sequential or cumulative, but more a patient phenotype, with stages 3 and 4 more frequently found in patients with low-flow low-gradient AS [26]. Nevertheless, there was a substantial and statistically significant increase in all-cause death and cardiac death at one year with each cardiac damage stage, so that all-cause death occurred in 24.5% of participants in stage 4 and in 21.3% in stage 3 [23].

#### 3.2.3. Right Ventricular (RV) Damage

RV dysfunction in this case also has a complex pathophysiology determined by both elevated filling pressures in AS and chronic volume overload caused by the ASD. Moreover, RV dysfunction is a prognostic factor in patients with severe AS, encountered in almost one third of patients [27]. Because of the prognostic impact, both PH and RV dysfunction were considered advanced stages of cardiac damage with significantly higher mortality rates even after TAVI [28]. A meta-analysis of 16 studies which included 14,499 patients with severe symptomatic AS and moderate/severe asymptomatic AS found that 16.1% of patients had stage 3 and 11.2% stage 4 cardiac damage and that these categories of patients had significantly higher 1-year mortality irrespective of the presence of AS related symptoms [25].

RV function is closely linked to LV systolic function, filling pressures, ventricular interdependence, and the effects of pulmonary arterial hypertension and reduced RV-pulmonary artery coupling [29]. On the other hand, in ASD the mechanisms of RV deterioration are different and primarily related to the chronic volume overload which are often hemodynamically well tolerated until late in life [30]. Concomitant elevated end-diastolic LV and LA pressures, in the presence of an undiagnosed ASD results in increased left-to-right shunting which produces offloading of the less compliant LV and a pressure overload on the RV [31].

### 3.3. Diagnostic Challenges and the Role of Multimodality Imaging

Echocardiography (TTE, TOE) is the first line diagnostic tool for diagnostic and quantification in all structural heart diseases, including both AS and ASD [32,33]. Guideline directed echocardiographic evaluation provides accurate diagnosis in most patients, but there are more complex cases that require supplementary techniques to refine diagnosis and guide management.

Multimodality imaging—integrating TTE, TOE, cardiac CT, and invasive hemodynamic assessment—was pivotal not only for defining the anatomical substrate, quantifying shunt magnitude, and excluding associated anomalies, but also for meticulous procedural planning. Pre-interventional imaging allowed accurate sizing of both the transcatheter aortic valve prosthesis and the atrial septal occluder device, while ensuring that anatomical conditions were optimal for safe implantation. In the context of TAVI, detailed CT evaluation of the aortic annulus area, perimeter, sinus of Valsalva dimensions, and coronary ostial heights was essential to select the appropriate valve size and confirm adequate coronary clearance. Similarly, three-dimensional TOE reconstruction provided precise delineation of the ASD morphology, fenestrations, and rim adequacy, ensuring optimal device selection and positioning during percutaneous closure [34].

Complex imaging techniques, including 3D TOE and cardiac CT, are necessary to accurately describe the defect if interventional device closure is considered. The most important feasibility criteria include stretched diameter of the ASD < 38 mm and sufficient rim of 5 mm, except towards the aorta [33].

Cardiac magnetic resonance (CMR) is rarely required, but may be useful for the assessment of RV volume overload, quantification of RV systolic function and pulmonary to systemic flow ratio or identification of other abnormalities such as pulmonary venous connections [35], although these parameters can also be assessed by cardiac CT.

### 3.4. Therapeutic Approach—Rationale for Staged Interventional Strategy

In this complex setting, the therapeutic sequence was guided by pathophysiological reasoning. The priority was to eliminate the dominant source of pressure overload—severe AS—since the high LV filling pressures promoted and magnified the left-to-right shunt through the ASD. TAVI was therefore performed first, aiming to normalize LV outflow and filling pressures, thereby allowing a more accurate assessment of the true hemodynamic burden imposed by the shunt. Despite marked early improvement in RV systolic performance after TAVI, the RV remained significantly dilated, highlighting the persistent impact of volume overload.

Moreover, despite the correction of AS, management needs to be refined not only by anatomical and feasibility criteria, but by invasive hemodynamic evaluation. RHC is mandatory prior to ASD closure, and especially after correcting a left heart valvular disease [31]. When the non-invasive calculated PAP exceeds 40 mmHg or other indirect signs of severe PH are present, RHC is required to determine pulmonary vascular resistances (PVR) [33]. The guideline indications for ASD closure are a ratio of pulmonary over systemic flow >1.5 and enlargement of the right-sided cavities, while severe PH (PVR > 5 WU) represents a contraindication for closure [33].

Only after confirming hemodynamic stability, euvolemia, and the absence of arterial PH, percutaneous ASD closure was pursued. This stepwise approach minimized the risk of acute rises in LA pressure and pulmonary congestion that could have occurred if the shunt were closed prematurely [9].

#### 3.4.1. TAVI in Patients with Heart Failure and Pulmonary Hypertension

TAVI has increasingly become the preferred therapeutic intervention in patients with severe AS, regardless of surgical risk, especially in the elder populations [36]. The coexistence of PH in TAVI candidates increases the hemodynamic burden, elevating RV load and impairing procedural stability. In an analysis from the FRANCE registry patients were divided into 3 subgroups according to estimated sPAP determined on ETT in no PH (sPAP < 40 mmHg), mild-to-moderate PH (sPAP 40–59 mmHg) and severe (sPAP > 60 mmHg) and even if short-term mortality was similar between subgroups, at one year mortality was significantly higher in the moderate and severe PH groups (29% vs. 22%) [37]; interestingly, the same analysis showed no difference in functional capacity at one year. Longer follow-up data on the prognostic impact of PH after TAVI are still scarce. Still, considering the complex mechanisms of PH in patients with AS, reduction of sPAP is often seen after LV and RV unloading after TAVI. Alushi et al. used a similar categorization of patients in no PH (sPAP < 34 mmHg), mild-to moderate PH (sPAP < 46 mmHg) and severe (sPAP > 46 mmHg); after TAVI, sPAP decresed significantly at discharge in 46% of patients and at one year the reduction was observed in most patients [38]. The conclusion was that even if PH is a predictor of increased mortality on longer follow-up after TAVI, patients with reversible PH are at lower risk of all-cause mortality and benefit from TAVI [38].

#### 3.4.2. Percutaneous ASD Closure in the Elderly

It is known that the best results of ASD closure are at a young age, ideally before 25 years [39]. After the age of 40 years, there is an increased likelihood of comorbidities and especially conditions that increase LV filling pressures, but still patients benefit from the intervention especially if it can be performed percutaneously [40].

In patients with impaired LV function (systolic or diastolic), such as the presented case, ASD may worsen heart failure. Comprehensive evaluation is required, in some cases effort testing or invasive balloon occlusion with reassessment of hemodynamics to decide between complete, fenestrated or no closure [31].

Although many ASD remain asymptomatic until late in life, patients with symptoms of HF due to RV overload may benefit from percutaneous closure. In a meta-analysis of 18 single-arm cohorts comprising of 1184 patients over the age of 60 years submitted to transcatheter ASD closure, statistically significant reduction of RV dilatation, sPAP, tricuspid regurgitation and BNP were noted, as well as an improvement in quality of life and functional capacity [41]. Moreover, considering the risk of worsening HF, a retrospective analysis following ASD closure in patients over 60 years showed a low rate of heart failure hospitalizations after ASD closure, in a median follow-up period of 4 years [42].

### 3.5. Heart Team and Complex Structural Heart Disease—Between Current Guidelines and Gaps in Evidence

The management of patients with coexisting valvular and congenital heart disease occupies a rare clinical space where evidence-based guidelines offer some direction, but no certainty. Available recommendations such as the 2025 ESC/EACTS guidelines for valvular heart disease, the 2020 ESC guidelines for adult congenital heart disease, and the 2022 ESC/ERS pulmonary hypertension guidelines address individual lesions in isolation, but do not provide an integrated algorithm for the patient who presents with all three conditions simultaneously [17,32,33]. The coexistence of degenerative severe AS and secundum ASD in the elderly represents an exceptionally rare clinical entity, with the published literature limited to isolated case reports treated surgically or with simultaneous transcatheter interventions [43,44,45]. As there are very limited evidence-based recommendations, the Heart Team role in the management of such patients is indispensable. In the present case, the Heart Team’s decision to stage the interventions—TAVI first, followed by reassessment of shunt significance and deferred ASD closure—was not derived from a guideline flowchart, but from a shared understanding multiple mechanisms causing HF and PH, but also the risks of premature shunt closure in a poorly compliant left ventricle.

Reports describing a staged transcatheter strategy in elderly patients with degenerative tricuspid AS and an unrecognized secundum ASD presenting with dual-mechanism pulmonary hypertension are exceedingly rare. The novelty of our report lies in the physiology-guided sequencing of TAVI, post-procedural invasive hemodynamic reassessment, and deferred ASD closure based on demonstration of reversible pulmonary hypertension and persistent clinically significant shunting. To our knowledge, this is one of the few detailed descriptions of a staged transcatheter approach—TAVI followed by percutaneous ASD closure—in an elderly patient with degenerative tricuspid AS and an unrecognized secundum ASD presenting with dual-mechanism pulmonary hypertension and heart failure. This case reinforces that in elderly patients with unexplained or disproportionate PH, RV dilatation, or mixed signs of pressure and volume overload, the possibility of an unrecognized concomitant congenital heart disease should be actively investigated. A multimodality imaging strategy and Heart Team–based decision-making remain cornerstones for achieving optimal outcomes in such challenging scenarios.

There are recognized gaps in evidence due to the minimal volumes of patients in each cardiac center, the lack of relevant outcome measures to ensure quality of care, and the lack of biomarkers and neurohormones to estimate disease severity and timing of interventions [33].

## 4. Conclusions

This case highlights the exceptional hemodynamic complexity arising from the coexistence of severe AS and a large ostium secundum ASD in an elderly patient. The overlapping pressure and volume overload imposed by these two lesions led to advanced RV remodeling and PH, with subsequent congestive HF, which were only partially reversible after the correction of left-sided obstruction. A comprehensive, stepwise strategy—initiating with optimal decongestion and guideline-directed medical therapy for HF, followed by TAVI and delayed percutaneous ASD closure—proved crucial for achieving both hemodynamic stabilization and sustained clinical improvement.

Multimodality imaging played a central role throughout the diagnostic and therapeutic course, ensuring accurate anatomical characterization, optimal device sizing, and procedural safety.

This case underscores the importance of early recognition of dual pathology and the value of a multidisciplinary, imaging-guided approach in tailoring interventions for patients with combined structural and valvular heart disease.

## Data Availability

The original contributions presented in this study are included in the article. Further inquiries can be directed to the corresponding author.
